# *Clostridium septicum* Aortitis of the Infrarenal Abdominal Aorta

**DOI:** 10.14740/jocmr2435w

**Published:** 2015-12-28

**Authors:** Aditya Shah, Tariq Yousuf, Mohammed Rachid, Naureen Ali, Muhammad Tabriz, Kevin Loughry

**Affiliations:** a Advocate Christ Medical Center, 4440 W. 95th Street, Oak Lawn, IL 60453, USA

**Keywords:** Aortitis, Colonic malignancy, *Clostridium septicum*

## Abstract

*Clostridium septicum* aortitis is a rare infection that has a strong association with occult colonic malignancy. There is also emerging evidence to support the combination of medical and surgical management over medical management alone. To the best of our knowledge, we report the 40th known case of *C. septicum* aortitis.

## Introduction

*Clostridium septicum* (*C. septicum*) can cause a wide array of clinical manifestations including gas gangrene. One proposed mechanism of infection is by hematogenous spread from the gastrointestinal tract. Gas gangrene caused by *C. septicum* is associated with colorectal cancer and other defects of the bowel. Alpern et al and Kornbluth et al have reported an association between *C. septicum* infection and malignancy [1, 2]. We describe a rare case of *C. septicum*-induced aortitis affecting the infrarenal abdominal aorta. We received consent from the patient for publication of this case.

*C. septicum* is a Gram-positive, spore forming, obligate, anaerobic bacterium. *C. septicum* causes myonecrosis through the release of exotoxins such as the alpha toxin [3], lethal toxin, and hemolytic toxin.

An infected aneurysm, also known as a “mycotic aneurysm” or “microbial arteritis”, is an aneurysm arising from bacterial infection of the arterial wall. It was described first by Osler in 1887 [4]. This complication is not all that uncommon and it is caused by the hematogenous spread of bacterial infection. Given that the current treatment modalities for aortic aneurysms may be time sensitive, early diagnosis is prudent. Without medical or surgical management, severe hemorrhage, rupture, or uncontrolled sepsis may occur [5]. Despite this, symptomatology is frequently nonspecific during the early stages so a high index of suspicion is required to make the diagnosis.

We present a case of a 78-year-old patient who was found to have an incidental aortitis in the setting of intussusception and colon cancer.

## Case Report

The patient was a 78-year-old male who presented with chronic diarrhea of 2 years’ duration. He initially presented to his gastroenterologist who performed a colonoscopy and discovered colonic cancer at the hepatic flexure. Pathology revealed moderately differentiated adenocarcinoma with complex focally cribriform glands formed by cells with enlarged hyperchromatic nuclei. The patient was then admitted to the hospital for further workup and staging of the malignancy.

On admission, the patient had a fever of 39.0 °C, heart rate of 96 beats/min, hemoglobin of 12 mg/dL, and leukocytosis of 17,000/μL. Tumor marker carcinoembryonic antigen was 137 μg/L.

**Figure 1:**
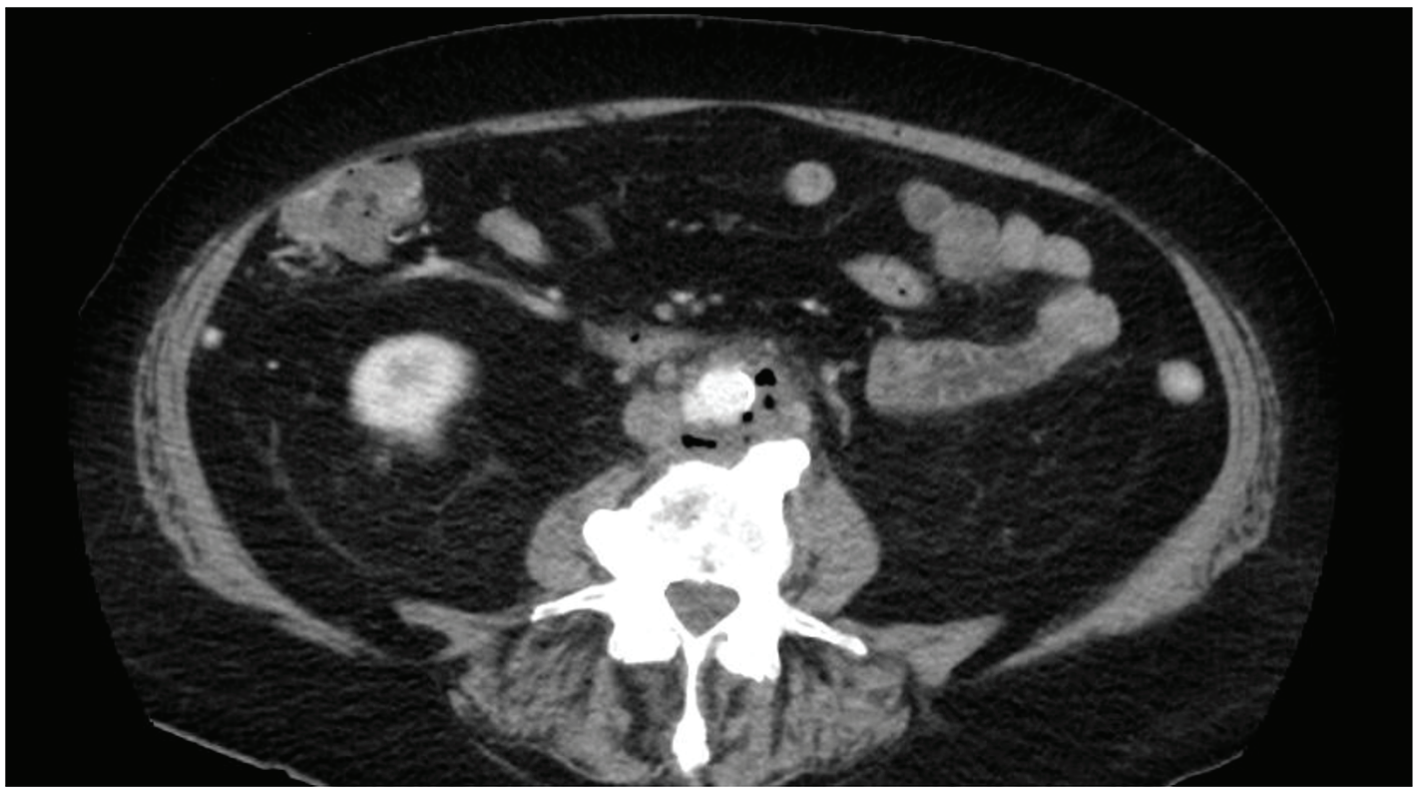
Coronal CT showing intussusception at the junction between the transverse colon and the hepatic flexure.

He underwent a CT scan for further malignancy staging and was found to have an intussusception at the location of his newly discovered colonic cancer (Fig. 1).

**Figure 2:**
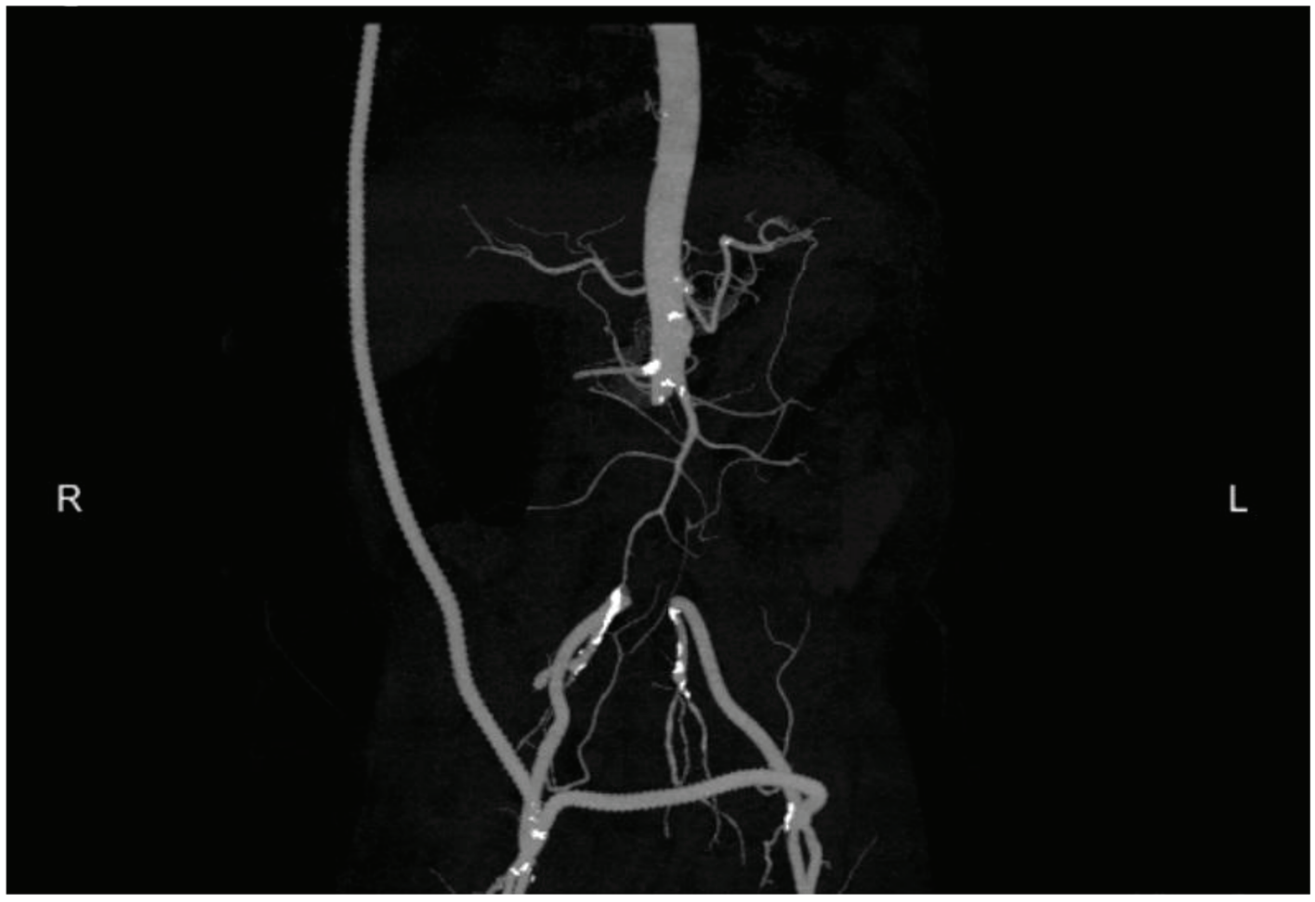
Aortitis with periaortic abscess and a pseudo aneurysm in the infrarenal abdominal aorta.

**Figure 3:**
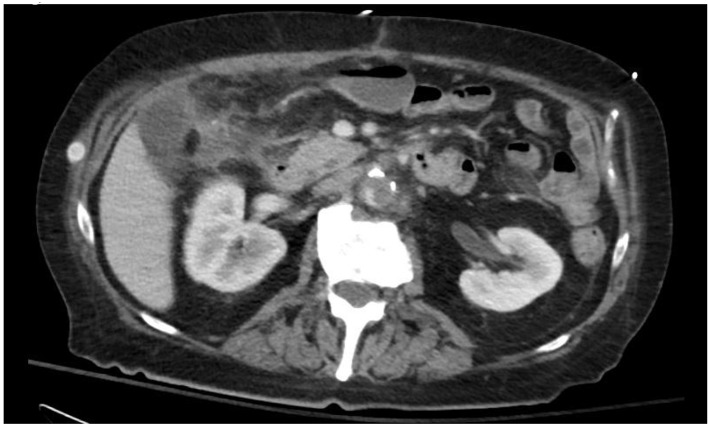
Coronal CT showing mycotic aneurysm of the infrarenal aorta with gas bubbles.

The CT scan also incidentally revealed findings consistent with aortitis with a periaortic abscess and an asymptomatic pseudoaneurysm in the infrarenal abdominal aorta (Fig. 2 and 3). Blood cultures also grew *C. septicum* which was pan-susceptible. On further questioning, the patient endorsed a 10 pound weight loss over the preceding few months with intermittent explosive diarrhea along with decreased appetite, weakness, and generalized malaise. The patient denied other constitutional symptoms such as fever or chills. Prompt resection of the infrarenal aorta was performed. Pre- and post-operatively the patient was started on intravenous aztreonam, vancomycin, and metronidazole and sent home on oral metronidazole. In the interim, he also underwent elective definitive management of his neoplasia with colon resection of the hepatic flexure mass.

The patient was doing well post-operatively for several months undergoing meticulous multi-disciplinary care until he started developing fevers, chills, and weakness. He presented back to the hospital and was found to have an abscess at the post-operative anastomotic site of the colon cancer resection at the hepatic flexure. Blood cultures were drawn which regrew *C. septicum*. He was managed with the same combination of intravenous antibiotics and was discharged home on oral metronidazole and levofloxacin. The plan was to continue the current antibiotics and to do a follow-up CT scan to confirm the response of the abscess to the proposed treatment.

## Discussion

A typical finding of clostridial mycotic aneurysms in the CT scan is gas formation surrounding the aorta or peripheral arteries. Clostridia can proliferate in tissues when oxidation-reduction falls or the pH is reduced, which may occur with arterial injury, necrotic tissue, or anoxic tissue with lactic acid accumulation [6]. For this reason, clostridial infection is frequently associated with gastrointestinal or hematologic malignancy. Kornbluth et al reported an associated malignancy in 81% of patients with *C. septicum* infection, which has been validated by other similar studies [2]. A study of human fecal flora in healthy volunteers showed that *C. septicum* is not normally present [7]. Only about 1.3% of clostridial infections are caused by *C. septicum* [8]. It is believed that ulcerative lesions of the gastrointestinal tract, especially colon carcinoma, can allow clostridial organisms to enter the bloodstream and seed an atherosclerotic focus in the aorta, resulting in the development of mycotic aortic aneurysm [9]. Therefore, the diagnosis of clostridial mycotic aortic aneurysm requires a thorough search for an occult malignancy.

Of the total 40 cases of aortitis caused by *C. septicum* that have been reported and listed in Table 1 [9-46], at the time of the review the aneurysm was located in the infrarenal aorta in 13 (34.2%), abdominal aorta (including juxtarenal and suprarenal) in nine (23.6%), the thoracic aorta (ascending part and the aortic arch) in 10 (26.3%), the iliac artery in three, the thoracoabdominal in two, the whole aorta in two, the popliteal artery in one, and the thoracic aorta and abdominal aorta (double aneurysm) in one patient. Of these 40 cases, seven cases experienced aortic dissection and one case experienced aortic rupture. Also, two cases were reported in young age (16 years old and 22 years old) and both of them ended up in aortic dissection and death. In these 40 cases, there were 30 cases (78.9%) with colon neoplasm. Twenty-two of the 24 patients who underwent vascular surgery survived (91.6% survival rate, 8.4% mortality rate), whereas four out of five cases that treated medically only died (80% mortality). Out of these 40 cases, 10 cases died before getting accurate diagnosis, diagnosed at autopsy, or did not make it till the time of the surgical intervention. Surgical treatment seems to be needed to achieve optimal results.

**Table 1:** Cases of Aortitis Caused by *C. septicum*

First author	Year	Age	G	Process	Location	Neoplasm	Intervention	Outcome
Bridges [10]	1981	68	M	Aortic aneurysm	Infrarenal abdominal aorta	No	Extra-anatomical bypass (axillobifemoral bypass) and omental flap	Alive
Semel [11]	1984	60	F	Aortic aneurysm	Aortic arch- ascending aorta	Transverse colon cancer	Colon resection only	Died of cardiac tamponade 20 h after colon resection
Kaufman [12]	1988	62	M	-	Bilateral iliac arteries and femoral arteries	None	None	Died
Narula [13]	1988	76	M	-	Right popliteal artery	Cecal cancer	Resection of the aneurysm	Alive
Momont [14]	1989	85	F	Aortic aneurysm and dissection	Dissection of the ascending aorta and the arch	Cecal cancer	None	Died (sepsis?)
Asplund [15]	1990	80	M	-	Right iliac artery	Cecal cancer	Extra-anatomical bypass (femorofemoral bypass)	Alive (late death due to liver metastasis
Skipper [16]	1990	70	F	Aortitis	Suprarenal Abdominal aorta	No	Diagnosed at autopsy	Died during surgery
Brahan [17]	1990	70	F	Aortic aneurysm	Aortic arch- descending aorta	Ascending colon cancer	*In situ* graft replacement, resection of a fistulas between the aneurysm and the pulmonary artery	Alive
Hurley [18]	1991	67	M	Aortic aneurysm	Infrarenal abdominal aorta	Colonic polyps (rectum, splenic flexure and cecum)	Rt. axillobifemoral bypass followed by resection of the aneurysm, It. Ax-F bypass was performed because of infection of the rt. Ax-F bypass	Alive 9 months postoperatively
Christensen [19]	1993	74	F	Aortic aneurysm	Juxtarenal abdominal aorta	-	None	Died
Messa [20]	1995	77	M	Double aortic aneurysm	Distal descending aorta and infrarenal abdominal aorta (double aneurysms)	Sigmoid polyp cancer	Extra-anatomical bypass (axillobifemoral bypass) for the abdominal aorta, and *in situ* graft replacement of the descending aorta and omental flap	Alive
Murphy [21]	1996	78	M	Aortic aneurysm	Proximal descending aorta	Sigmoid polyps	*In situ* graft replacement, esophagectomy	Alive after 6 months
Sailors [22]	1996	74	F	Aortic aneurysm	Thoracoabdominal aorta	-	*In situ* replacement	Died presumably due to rupture of pseudoaneurysm in the distal anastomosis
Monsen [23]	1997	81	M	Aortic dissection	Dissection of the whole aorta (rupture at the infrarenal abdominal aorta	Cecal cancer	*In situ* graft repair	Died 6 h after surgery
Montoya [24]	1997	78	M	Aortic aneurysm	Descending aorta	Cecal cancer	None	Died 16 h after admission
Cohen [25]	1998	77	M	Aortic dissection and abscesses	Aortic root and Ascending aorta	Cecal adenocarcinoma	Rt. hemicolectomy	Died 23 days post-op
Johnson [26]	1999	78	M	Aortic aneurysm	Infrarenal abdominal aorta	-	None	Died 6 days after admission
Morrison [27]	2001	71	M	Aortic aneurysm	Thoracoabdominal aorta	Ascending colon cancer	*In situ* graft replacement	Alive
Al Bahrani [28]	2001	63	M	Aortic aneurysm	Infrarenal abdominal aorta	Ascending colon cancer	*In situ* graft replacement?	Alive?
Zenati [29]	2002	87	M	Aortic aneurysm and dissection	Abdominal aorta	Cecal adenocarcinoma	None	Died in the hospital at day 6
Munshi [30]	2002	78	M	Aortic aneurysm	Infrarenal abdominal aorta	Cecal adenoma	None	Died 1 month after discharge
Takano [31]	2003	69	M	Aortic aneurysm	Infrarenal abdominal aorta	Ascending colon cancer	*In situ* graft replacement, rectus abdominal muscle flap	Alive
Liechti [32]	2003	55	M	Aortic aneurysm	Infrarenal aorta	Transverse colon adenocarcinoma	Transverse colectomy and exploration of the aorta without resection	Died 5 months after admission
Davies [33]	2003	63	M	Aortic aneurysm	Infrarenal	Unknown	Axillobifemoral bypass	Died 2 days post-op
Rucker [34]	2004	77	F	Aortic aneurysm	Infrarenal	Cecal adenocarcinoma	Axillobifemoral bypass and right colectomy	Died 42 days post-op
Rucker [34]	2004	91	F	Aortitis	Abdominal aorta	Ascending adenocarcinoma	Rt. hemicolectomy	Unknown
Evans [35]	2004	91	F	Aortitis	Abdominal	Transverse colon adenocarcinoma	Extended Rt. hemicolectomy	Died at 5 months
Creed [36]	2004	77	F	Aortitis then Aneurysm	Infrarenal Aneurysm	Colon cancer, poorly differentiated adenocarcinoma	Rt. hemicolectomy + axillobifemoral by pass + resection of infrarenal and two common Iliac arteries	Died on the 42nd POD 2/2 sepsis and MOF
Mohamed [37]	2006	82	M	Aortic aneurysm	Juxtarenal	Ascending adenocarcinoma	*In situ* graft, Rt. hemicolectomy	Alive
Asciutto [38]	2007	71	M	Aortitis then Aortic rupture	Juxtarenal Abdominal aneurysm	Colon carcinoma, ascending colon	Rt. hemicolectomy, then aortic replacement with dacron tube + IV Abx	Alive
Seder [9]	2008	75	M	Aneurysm	Infrarenal	Ascending adenocarcinoma	Rt. hemicolectomy and axillobifemoral bypass	Died at 4 months due to recurrent aortitis
Seder [9]	2008	76	F	Aortic aneurysm	Juxtarenal	Cecal adenocarcinoma	Axillobifemoral bypass and right hemicolectomy	Died at 94 day post-op
Yang [39]	2009	22	M	Aortitis then aortic dissection	Whole length of aorta	No	Nothing was done, diagnosed at autopsy	Died
Eplinius [40]	2010	32	M	Aortic dissection	Thoracic aorta	No	Diagnosed at autopsy	Died
Moseley [41]	2010	82	M	Aortitis	Infrarenal and Rt. common iliac artery	Cecal tubulovillous adenoma (high grade)	Suppressive IV Abx + Rt. hemicolectomy (Zosyn, Vanc and Levoflox) then IV cefepime and metronidazole (Pt. refused surgery)	Survival for 75 days after admission and died 2/2 ischemic heart dis.
Tsukioka [42]	2013	74	M	Aortic aneurysm *C. difficle*	Rt. common iliac artery	No	Excision of infrarenal and both common Iliac arteries	Alive
Lintin [43]	2014	78	F	Aortic aneurysm	Arch of aorta and thoracic aorta	Cecal adenocarcinoma and hepatic metastasis	Hybrid endovascular repair + laparoscopic Rt. hemicolec. + liver metastasis resection	Alive
Al Hadi [44]	2014	63	M	Aortic aneurysm	Aortic arch to mid thoracic aorta	Colorectal cancer	Rt. hemicolectomy and he did not make it to the vascular surg.	Died before the vascular surgery
Subramaniam [45]	2014	16	F	Aortic dissection	Ascending thoracic aorta	No	Diagnosed at autopsy	Rapid course of events, died before diagnosis
Shah [46]	2015	78	M	Aortic aneurysm	Infrarenal aorta	Colon cancer, hepatic flexure	Infrarenal resection and bypass grafting + IV Abx	Alive

The traditional surgical dictum mandates excision of the infected aneurysm, wide local debridement, administration of antibiotics, and remote grafting in the form of extra-anatomic bypass through a clean surgical field. However, *in situ* reconstruction has received emphasis in recent years. In the presence of a positive gram stain or purulence, excision of the pseudo aneurysm with an extra-anatomic bypass should be used, followed by a 6-week course, at the minimum, of parenteral antibiotics. In the absence of purulence and with a negative gram stain, *in situ* graft reconstruction with synthetic material can be utilized, followed by a 6- to 8-week course of organism-specific antibiotics [20].

Our patient had an emergent right axillofemoral bypass with an 8 mm heparin bonded polytetrafluoroethylene (PTFE). Abdominal aortic exploration and ligation of the infrarenal abdominal aorta was performed with aortic debridement. Thrombectomy of the right axillofemoral bypass was also performed and the peripheral perfusion was left intact. As per current treatment guidelines, the patient then received long-term parenteral antibiotics with aztreonam, vancomycin and metronidazole [9]. He was eventually transitioned to only oral metronidazole and continued to clinically improve up until the discontinuation of antibiotics.

### Conclusion

Our literature review revealed that surgical and medical management (8.4% mortality rate) was superior to medical management alone (80% mortality rate) in patients with this rare condition which is summarized in Table 2. Consideration of immediate surgical management in addition to medical management of these patients should be given.

**Table 2:** Summary of Surgical and Medical Management

Total number of patients with reported *Clostridium aortitis*	40
Males	27
Females	13
Average reported age	70.5 years old
Aortic injury	
Aortic aneurysm alone	25 (62.5%)
Aortic dissection	7 (17.5%)
Aortitis alone	4 (10.0%)
Aortic rupture	1 (2.5%)
Unknown process	3 (7.5%)
Interventions	
Surgical and medical Rx	24 (60.0%)
Medical Rx only	5 (12.5%)
None	11 (27.5%)
Mortality rate following intervention	
Surgical and medical Rx	2 out of 23 (8.6%)
Medical Rx only	4 out of 5 (80%)
None	12 out of 12 (100%)
Total reported survivors	22 (55.0%)
Total reported mortalities	17 (42.5%)
Unknown outcome	1 (2.5%)

Owing to the rare nature of the condition, and most cases being managed with a combination of medical and surgical management, one limitation of our conclusion could be that the number of patients treated only medically might be too small to make a confirmed recommendation of the treatment goals and standards going forward.

In conclusion, our case is one of few reported cases of aortic aneurysm related to clostridium bacteremia. Most of the reported cases in the literature have been associated with colonic malignancy like in our patient. Hence, it would be prudent for clinicians to do a thorough search for malignant processes in patients presenting with similar complaints and *C. septicum* bacteremia, and also give serious consideration to prompt surgical management of the same.
